# Towards Individually Tailored Diets in Prevention and Treatment of Diabetes?

**DOI:** 10.3390/nu15122649

**Published:** 2023-06-06

**Authors:** Matti Uusitupa, Ursula Schwab

**Affiliations:** 1 Institute of Public Health and Clinical Nutrition, School of Medicine, University of Eastern Finland, 70211 Kuopio, Finland; 2 Finland and Department of Medicine, Endocrinology and Clinical Nutrition, Kuopio University Hospital, 70211 Kuopio, Finland

Type 2 diabetes (T2D) is a heterogenous disease regarding its phenotype and genotype. The huge increase in the prevalence of T2D in the last 40 years worldwide strongly indicates that the epidemic of T2D is mainly due to our lifestyles, i.e., increasing prevalence of obesity along with sedentary lifestyles and unhealthy dietary habits. The heritability of T2D is strong, ranging from 30 to 70%, and genetic factors are involved in the diverse pathophysiology of T2D. Currently, 700 genetic markers have been identified to increase the risk of T2D. This Special Issue of *Nutrients* focuses on the genes and gene and diet interactions that may modify or change the principles of general advice in the dietary management of T2D and its preceding conditions, impaired fasting glucose, and impaired glucose tolerance. The purpose is to update our knowledge of where we truly are now in precision medicine regarding the dietary treatment of T2D [1]. One of the focuses of this Special Issue is the genetics of gestational diabetes, whose prevalence has also increased substantially in recent decades along with the epidemic of T2D. Gestational diabetes has increased markedly, e.g., hypertensive pregnancy disorders and fetal macrosomia, and in the long run, the risk of T2D and cardiovascular morbidity, as well as the risk of type 1 diabetes, is increased in mothers with former gestational diabetes [2].

This Special Issue includes three other papers dealing with the study design and feasibility of randomized studies on the effect of group and Internet-based counseling interventions on the incidence of T2D and deterioration of glucose metabolism in groups with high vs. low genetic risk scores for T2D [3]; a review on the gut-microbiota-derived metabolite indole propionic acid [4]; and a secondary analysis on metabolites and gene expression data obtained from a fish protein intervention study with an eight-week duration [5]. We hope the studies in this Special Issue will stimulate interest in precision medicine regarding nutrition.

In the review article on the genetics of T2D, the authors describe the history and huge and rapid progress of genetic studies of T2D, focusing on a more detailed genetic classification of T2D based on modern GWAS studies. One of the problems in the classification of T2D into subgroups is the phenotype, which may also change with time. The first identified genetic risk locus based on the candidate gene approach was the PPARG2 genetic variant that was associated with T2D risk, obesity, and insulin resistance. In 2006, the discovery of the TCF7L2 gene variant that is involved in insulin secretion represented remarkable progress in genetic studies on T2D. The genetic variant (rs7903146) increases the risk of T2D by 41% [1]. Soon after the observation of TCF7L2’s link with T2D, analyses based on two lifestyle prevention trials showed that the increased risk of T2D linked to this genetic variant can be overcome by weight loss and increased exercise [6,7]. The first GWAS study on the genetics of T2D was published in 2007. Since then, numerous GWAS studies have been carried out with an increasing number of study individuals and various phenotypes of T2D and other glucose metabolism disturbances. Altogether, an increasing number of genetic loci have been identified to be associated with T2D phenotypes, and the vast majority of them are linked with insulin secretion. What are the clinical implications of these findings? Regarding maturity-onset diabetes of the young (MODY), genetic studies are mandatory to classify the disease form exactly, and knowing the genetic variant helps to tailor the most suitable treatment modality for a particular subtype of MODY, as the authors emphasize in the conclusions [1].

There has been great progress in understanding the pathophysiology of gestational diabetes during the era of modern genetics [2]. The article “Genetic Risk Factors and Gene–Lifestyle Interactions in Gestational Diabetes” provides a very comprehensive view on the current progress in the genetics of gestational diabetes, which, along with T2D, is a heterogenous disease in terms of its phenotype and genotype. Currently, there are few implications to use genotyping of gestational diabetes since solid trial evidence is lacking when it comes to the selection of different treatment modalities of this disease based on the genetic data. It is not a surprise that the well-known risk variants of T2D are also involved in the pathogenesis of gestational diabetes, but also unique variants for gestational diabetes have been identified. The authors point out that the effect of a single variant is commonly low, as is the case with T2D. They conclude that more studies are needed to identify the impact of rare variants on the risk of gestational diabetes. In this article, there is an interesting speculation on what the effect of paternal, placental, and fetal (genetic) factors is on the later prognosis of both the fetus and mother. Genetic risk scores have also been applied in the diagnostic tools of subtypes of gestational diabetes, and there is some evidence that high-risk mothers may benefit more from lifestyle modifications, i.e., healthy dietary changes and increasing physical activity, than mothers with a lower genetic risk [2].

The Finnish T2D-GENE study, whose study design and results of applicability and feasibility are reported in the present Special Issue, is an example of a gene x lifestyle intervention study where genetic identification of individuals is carried out before the study [3]. The aim of the present report was to examine whether group and Internet-based counseling on healthy diet and physical activity works in the real world. The feasibility and adherence to lifestyle changes in the intervention groups were confirmed by the number of website logins and self-reported physical activity. Furthermore, in the intervention participants, fiber intake increased, the quality of fat improved, and salt intake decreased based on calculations from the repeated 4 d food records during the entire study with a 3-year duration. The main aim of the T2D-GENE study is to examine whether individuals with a high genetic risk score for T2D based on 76 risk variants have different responses to lifestyle interventions as compared to those with a low genetic risk, the main outcome measure being incident diabetes. This study is the first randomized trial primarily planned to resolve this question. Earlier observations are based mainly on post hoc analyses of former interventions. Besides incident diabetes, changes in glucose tolerance, insulin secretion, and insulin sensitivity indices were also included in the outcome measures [3].

Metabolomics has become one of the key interests in studies on T2D pathophysiology. Many of the numerous metabolites connected to T2D risk reflect both genetic and environmental effects. Furthermore, the gut microbiota seems to play an important role in the pathogenesis of T2D. There are an increasing number of publications on associations between gut-derived metabolites (e.g., short-chain fatty acids, certain amino acids, bile acids, odd-chain fatty acids) and glucose metabolism. Most metabolites are associated with insulin resistance. Interestingly, the gut-derived tryptophan metabolite indole propionic acid (IPA) has been reported to be related to insulin secretion, and it may have many other biological effects outside of the gut. Interestingly, high intake of dietary fiber may exert some of its beneficial effects by increasing the IPA concentration in the blood. One of the papers published in this Special Issue focuses on the effects of IPA on glucose metabolism and non-alcoholic fatty liver disease [4].

The fish meat study in this Special Issue reports the metabolomics and gene expression data obtained from an eight-week intervention study with 5.2 g/d of salmon protein. Salmon protein had no effect on glucose metabolism [5]. The authors report metabolomics and gene expression data analyzed from peripheral blood mononuclear cells of the study participants, comparing the data of individuals with a high vs. low insulin response (AUC values) in a 2 h oral glucose tolerance test. Compared to the low-insulin-response group, the high-insulin-response group had higher plasma concentrations of monounsaturated fatty acids (MUFAs) and glycated protein A (GlycA), and lower concentrations of glycine and acetate, but the salmon protein intervention did not modify these metabolites, except for acetate. No statistically significant differences between the high- and low-insulin-response groups were observed in gene expression when accounting for multiple comparisons. The statistical power was limited due to the small number of study participants [5].

Precision medicine and precision nutrition in the prevention and management of T2D have gained a lot of interest within the last few years. The key question is whether the heterogeneous nature of T2D implicates more specific treatment modalities. Current evidence suggests that weight reduction, healthy dietary choices, and physical activity work in the prevention and remission of T2D associated with overweight and obesity, regardless of the genetic background. The identification of rare variants of T2D, comprehensive metabolomics data, and increasing knowledge of the health effects of gut microbiota, however, open new opportunities to more tailored dietary modalities in some subgroups of T2D, but these individually tailored treatment options should be tested in well-designed and controlled studies with the number of study participants ensuring statistical power. Furthermore, dietary instructions for T2D should be feasible to apply and promote the cardiovascular and general health of people with diabetes [8].

## References

[1] Laakso M., Fernandes Silva L. (2022). Genetics of Type 2 Diabetes: Past, Present, and Future. Nutrients.

[2] Jääskeläinen T., Klemetti M.M. (2022). Genetic Risk Factors and Gene-Lifestyle Interactions in Gestational Diabetes. Nutrients.

[3] Schwab U., Lankinen M., Uusitupa M., Laakso M. (2023). Lifestyle Intervention Guided by Group and Internet-Based Counseling in the T2D-GENE Trial Supports Its Applicability and Feasibility. Nutrients.

[4] Sehgal R., de Mello V.D., Männistö V., Lindström J., Tuomilehto J., Pihlajamäki J., Uusitupa M. (2022). Indolepropionic Acid, a Gut Bacteria-Produced Tryptophan Metabolite and the Risk of Type 2 Diabetes and Non-Alcoholic Fatty Liver Disease. Nutrients.

[5] Canet F., Christensen J.J., Victor V.M., Hustad K.S., Ottestad I., Rundblad A., Sæther T., Dalen K.T., Ulven S.M., Holven K.B. (2022). Glycated Proteins, Glycine, Acetate, and Monounsaturated Fatty Acids May Act as New Biomarkers to Predict the Progression of Type 2 Diabetes: Secondary Analyses of a Randomized Controlled Trial. Nutrients.

[6] Florez J.C., Jablonski K.A., Bayley N., Pollin T.I., de Bakker P.I., Shuldiner A.R., Knowler W.C., Nathan D.M., Altshuler D., Diabetes Prevention Program Research Group (2006). TCF7L2 polymorphisms and progression to diabetes in the Diabetes Prevention Program. N. Engl. J. Med..

[7] Wang J., Kuusisto J., Vänttinen M., Kuulasmaa T., Lindström J., Tuomilehto J., Uusitupa M., Laakso M. (2007). Variants of transcription factor 7-like 2 (TCF7L2) gene predict conversion to type 2 diabetes in the Finnish Diabetes Prevention Study and are associated with impaired glucose regulation and impaired insulin secretion. Diabetologia.

[8] The Diabetes and Nutrition Study Group (DNSG) of the European Association for the Study of Diabetes (EASD) (2023). Evidence-based European recommendations for the dietary management of diabetes. Diabetologia.

